# Evaluation of systemic inflammation in patients being weaned from mechanical ventilation

**DOI:** 10.6061/clinics/2018/e256

**Published:** 2018-06-12

**Authors:** Soraia Genebra Ibrahim Forgiarini, Darlan Pase da Rosa, Luiz Felipe Forgiarini, Cassiano Teixeira, Cristiano Feijó Andrade, Luiz Alberto Forgiarini, Elaine Aparecida Felix, Gilberto Friedman

**Affiliations:** I Fisioterapia, Centro Universitario – IPA, Porto Alegre, RS, BR; II Biomedicina, Faculdade Cenecista, Bento Gonçalves, RS, BR; III Centro Universitario Ritter dos Reis, Porto Alegre, RS, BR; IV Hospital Moinhos de Vento, Porto Alegre, RS, BR; V Hospital de Clinicas de Porto Alegre, Porto Alegre, RS, BR; VI Programa de Pos-Graduacao em Ciencias Pneumologicas, Universidade Federal do Rio Grande do Sul, Porto Alegre, RS, BR

**Keywords:** Mechanical Ventilation, Weaning, Inflammatory Factors

## Abstract

**OBJECTIVES:**

The aim of this study was to evaluate systemic inflammatory factors and their relation to success or failure in a spontaneous ventilation test.

**METHODS:**

This cross-sectional study included a sample of 54 adult patients. Demographic data and clinical parameters were collected, and blood samples were collected in the first minute of the spontaneous ventilation test to evaluate interleukin (IL)-1β, IL-6, IL-8, and IL-10, tumour necrosis factor alpha (TNFα) and C-reactive protein.

**RESULTS:**

Patients who experienced extubation failure presented a lower rapid shallow breathing index than those who passed, and these patients also showed a significant increase in C-reactive protein 48 hours after extubation. We observed, moreover, that each unit increase in inflammatory factors led to a higher risk of spontaneous ventilation test failure, with a risk of 2.27 (1.001 – 4.60, *p*=0.049) for TNFα, 2.23 (1.06 – 6.54, *p*=0.037) for IL-6, 2.66 (1.06 – 6.70, *p*=0.037) for IL-8 and 2.08 (1.01 – 4.31, *p*=0.04) for IL-10, and the rapid shallow breathing index was correlated with IL-1 (r=-0.51, *p*=0.04).

**CONCLUSIONS:**

C-reactive protein is increased in patients who fail the spontaneous ventilation test, and increased ILs are associated with a greater prevalence of failure in this process; the rapid shallow breathing index may not be effective in patients who present systemic inflammation.

## INTRODUCTION

The process of discontinuing ventilatory support is referred to as weaning from mechanical ventilation (MV) and occupies approximately 40% of the total time spent on MV 1,2. The most commonly used weaning methods are the spontaneous ventilation test (SVT) and the use of low support pressures, both of which reduce the duration of MV and consequently seem to reduce mortality 2. However, the test used to interrupt MV can result in cardiopulmonary stress and weaning failure 3, since the transition from MV to spontaneous breathing results in increased respiratory muscle energy requirements according to the degree of respiratory overload (respiratory stress) and the resulting increased oxygen demand, which lead to increased cardiac output (cardiovascular stress) 4. The cardiopulmonary stress imposed by the weaning process induces systemic inflammation through different biological systems associated with the sympathetic nervous system and the release of catecholamines, which can influence the transition process to spontaneous breathing 5,6,7.

A number of studies on the weaning process have established a relation between the use of protocols and the success of this process 8-11. Different protocols are used to clinically evaluate cardiorespiratory conditions during the SVT in order to predict the success or failure of this process 8. However, the relation between the success or failure of MV weaning and inflammation is unclear. Sellares et al. 5 evaluated the inflammatory process during the SVT and observed an increase in the production of IL-6 in patients in whom weaning failed.

Given that the SVT is stressful, our hypothesis was that the SVT would be associated with the intensity of systemic inflammation and thus to a greater probability of failure. Thus, we decided to analyse the inflammatory blood markers interleukin (IL)-1β, IL-6, IL-8, IL-10, TNF-α and C-reactive protein (CRP) in patients submitted to a weaning protocol and to evaluate the relation between these markers and failure in this process.

## METHODS

### Patients and the mechanical ventilation weaning protocol

This prospective study was conducted between February and December 2012 at the Intensive Care Unit (ICU) of Hospital Moinhos de Vento in Porto Alegre, RS, Brazil. All patients intubated and mechanically ventilated for more than 48 hours were consecutively included in this study if they fulfilled the following criteria: improvement or resolution of the causes that led to acute respiratory insufficiency (ARI); 2) lack of fever (>38°) or hypothermia (<35°); 3) haemoglobin above 9 g/dL; 4) satisfactory neurological status on the Glasgow Coma Scale (≥8) and/or the Richmond Agitation-Sedation Scale (between -2 and 0) after a pause in sedation and 5) arterial oxygen pressure (PaO_2_) above 60 mmHg with an inspired oxygen fraction (FiO_2_)≤0.4 and positive end-expiratory pressure between 5 – 8 cmH_2_O. The exclusion criteria were as follows: 1) tracheostomy; 2) patients on haemodialysis; 3) neurological alterations; 4) polytrauma; 5) upper digestive haemorrhage; 6) lack of cooperation; 7) decision to limit treatment to life support; and 8) ventilation mode changed to pressure support ventilation, given a PS≤14 cmH_2_O.

This study was approved by the Hospital Moinhos de Vento Research Ethics Committee, and because it incurred no changes to clinical practice and due to the observational nature of the study, the Hospital Moinhos de Vento ICU Informed Consent Form was used.

### Study protocols

The rapid shallow breathing index (RSBI) and maximal inspiratory pressure (PImax) were measured in patients meeting the inclusion criteria, and patients presenting RSBI<105 and PImax>-25 cmH_2_O were considered eligible for the weaning protocol.

The SVT was conducted for 30 minutes using a T-piece with supplementary oxygen therapy at 5 L/min. SVT failure was characterized as a return to invasive ventilatory support within 48 hours of extubation due to the presence or persistence of one of the following factors: 1) respiratory frequency (RF) above 35 breaths per minute; 2) peripheral oxygen saturation (SpO_2_) below 90% with FIO_2_≤0.4; 3) heart rate (HR)>140 and <50 beats per minute; 4) systolic blood pressure>180 or <70 mmHg; 5) decreased consciousness, agitation or sweating or 6) paradoxical chest wall motion.

If no signs of SVT failure were evident after 30 minutes, the patient was extubated. In case of SVT failure, the patient was reconnected to the mechanical ventilator.

### Data collection and definitions

All clinical and chart data relevant to the study were collected at the beginning and end of the protocol. Patients fulfilling the inclusion criteria for the weaning protocol underwent spirometry and manovacuometry.

Respirometry was conducted with a digital pneumotachometer (Ventronic II^®^- DHD Healthcare, Wampsville, NY, USA), which was connected to the patient’s orotracheal tube (OTT); the patient was then instructed to breathe normally through the equipment.

Likewise, manovacuometry was performed with an analogue vacuum manometer (Globalmed^®^ model M120, Porto Alegre, Brasil). The manometer was connected to the patient’s OTT with a unidirectional valve, and the readings from five subsequent inspiratory breaths were annotated. The highest value obtained among the five breaths was considered to be the PImax.

### Analysis of inflammatory factors

An arterial blood sample was collected for the analysis of arterial gasometry and CRP, whereas a venous sample was collected to analyse inflammatory factors during the first minute of the SVT. Blood was collected to evaluate arterial gasometry and CRP once again 48 hours after extubation for patients with a successful SVT or at the moment of test failure for patients without a successful SVT.

For inflammatory factor analysis, blood samples were centrifuged at 2500 rpm at 4° C for 20 minutes. The sera were then stored in sterile plastic Eppendorf tubes at -80° C for subsequent analysis. Cytokine levels were determined with the ELISA method (Ready-Set-Go!, eBioscience, San Diego, CA, USA). The following cytokines were evaluated: IL-1β, IL-6, IL-8, IL-10 and TNF-α, all of which were expressed in pg/mL, as described previously 5.

### Statistical analysis

The convenience sample totalled 54 patients. Statistical analysis was performed with SPSS 18.0 (SPSS, Chicago, IL, USA). Data normality was evaluated with the Shapiro-Wilk test. The numeric variables were expressed as the mean±standard deviation, and the categorical variables were expressed as the absolute value and percentage. Student’s *t*-test was used to compare variables. The Pearson correlation test was used to evaluate the correlation between inflammatory variables and CRP. Given that the IL levels were distributed asymmetrically, these levels were transformed into a logarithm and analysed with a *t*-test to compare outcomes. A Cox regression model was used to estimate the factors associated with SVT failure. The significance level adopted was 5% (*p*<0.05).

## RESULTS

The general clinical characteristics of the 54 patients included in the study are summarized in Table 1. In this population, 34 (63%) passed the SVT and 20 (37%) failed.

Table 2 shows the physiological parameters of the patients at the beginning of the SVT, indicating who passed and who failed. When the demographic variables were compared based on PImax, arterial gasometry, PaO_2_/FIO_2_ and cumulative water balance, no significant differences were observed. However, a significant reduction (*p*=0.03) was observed between the RSBI of the group that failed the SVT and the group that passed. Patients included in the study did not use vasopressors.

**Figure 1:**
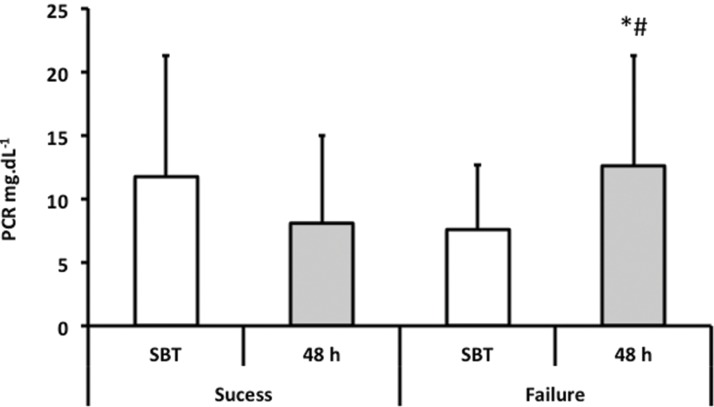
Evaluation of C-reactive protein in patients with success or failure in SVT. * - *p*=0.032 - comparison of SVT *vs*. 48h in the failure group; # - *p*=0.039 – comparison of 48h success *vs*. 48h in the failure group;

During the first minute of the SVT, there were no differences in CRP concentrations between the patients who failed and those who passed the weaning process. After 48 hours, the CRP levels of patients who failed were significantly higher than those during their first minute of the SVT, which is unlike the CRP levels in patients who passed the test (Figure 1).

The blood levels of IL-1β, IL-6, IL-8, IL-10 and TNFα were similar in both groups (Table 3). However, when the RSBI was evaluated with these factors, it was observed that for each unit increase in inflammatory factors, there was an increase in the risk of SVT failure [risk of 2.27 (1.001 – 4.60, *p*=0.049) for TNFα, 2.23 (1.06 – 6.54, *p*=0.037) for IL-6, 2.66 (1.06 – 6.70, *p*=0.037) for IL-8 and 2.08 (1.01 – 4.31, *p*=0.04) for IL-10].

Blood CRP levels correlated with IL-10 levels (r=0.91; *p*<0.05), as did RSBI values with IL-1 blood levels (r=0.51; *p*<0.05).

## DISCUSSION

The main finding of this study was that patients who failed the SVT presented higher inflammatory parameters than those who passed: 1) the IL levels were higher in patients who failed the SVT, but only IL-6 levels were significantly different between the two groups; 2) CRP levels increased after 48 hours in patients who failed the SVT; and 3) higher levels of TNFα, IL-6 and IL-8 were related to a higher incidence of SVT failure.

To the best of our knowledge, the relation between inflammatory markers and SVT failure has been infrequently studied and is poorly understood. The relationship between inflammation and the SVT seems to be partly related to the cardiopulmonary stress of patients during this process 15. The transition process from MV to spontaneous breathing could be considered a stress factor due to increased muscle energy demand and a consequent increase in oxygen consumption, which results in increased cardiac output. This cardiorespiratory stress could activate different response systems, including perhaps the sympathetic nervous system. Activation of this system would result in increased plasmatic catecholamines, such as IL-6 17. We observed significantly higher IL-6 serum concentrations in patients who failed the SVT than in patients who passed it. This result is similar to that of Sellares et al. 5, who observed higher IL-6 after the SVT than the moment before it. Except for IL-6, we found no significant differences among the other measures in patients who passed or failed the SVT.

In our study, we observed a 37% failure rate on the spontaneous breathing test; a similar figure was found in the literature, in which failure rates between 20 and 30% have been reported 2,16.

CRP analysis 48 hours after the SVT showed a significant increase in this marker both in patients who failed the SVT and in patients who passed. CRP is an acute phase reactant, and its plasma level increases four to six hours after a stimulus, presenting peak concentrations at 36 hours 17. We suppose that the intensity of the inflammation is due to cardiovascular stress, such as what occurs in acute exercise. Exercise induces increased IL-6 levels, which could stimulate increased CRP, reflecting the intensity of the inflammation process 18. However, a transitory increase in IL-6 induced by muscular force for a period of 30-60 minutes seems to have an inhibiting effect on pro-inflammatory cytokine activity, particularly on TNFα and IL-1 activity. IL-6 could trigger a cascade of anti-inflammatory cytokines and receptors, particularly IL-10 and receptors TNFα and IL-1, which could subsequently neutralize these potent pro-inflammatory cytokines 19. The study by Toumpanakis et al. hypothesized that inspiratory resistive breathing (IRB) results in increased mechanical stress, causing pulmonary inflammation in previously healthy lungs 20. The authors demonstrated that cytokines (IL-1 and IL-6) induced MMP-9 expression. In another study, the authors investigated the differential effects of IRB, expiratory resistance breathing (ERB) and combined resistive breathing (CRB). They demonstrated that CRB load dependently deranges mechanics, increases permeability, and induces inflammation in healthy rats 21. This finding could be one possible explanation for the results obtained by this group, as patients who failed the SVT presented high IL-6 and increased CRP with no other statistically significant differences among the other evaluated ILs.

The correlation observed between CRP and IL-10 is extremely interesting. The inhibitory function of IL-10 on the production of IL-12 and TNFα by means of activated macrophages and its inhibition of the expression of co-stimulators makes IL-10 an important component of homeostatic control in innate immune reactions and cellular immunity. Thus, increased CRP, an agent related to increased inflammatory response, could be associated with an increase in organic defence by means of IL-10 22.

We observed, moreover, an inverse correlation between RSBI and IL-1, which demonstrates that the higher the levels of systemic IL, the greater the RSBI of patients who undergo the SVT, clearly showing the influence of inflammation in this process. Although the inflammatory response is definitively present at the transcriptional level, it is inconsistently associated with an increase in the cytokine concentration of bronchoalveolar lavage, and moreover, it is not associated with changes in pulmonary physiological parameters, which could be related to evaluation duration, i.e., less than the six hours, as demonstrated in the abovementioned studies 23.

IL are a diverse group of inflammatory mediators produced by many types of cells, and they initiate and orchestrate responses to different stress factors, such as bacteraemia, shock and thermal injuries. IL act on different types of cells and can affect all organs in the critically ill from both a physiological and biochemical point of view, and they interact with different types of cellular receptors. Although this interaction occasionally involves a series of intracellular signals that result in the synthesis of proteins and IL, such as white blood cells, this process is not regulated and can result in amplification of the inflammatory cascade and uncontrolled activation in immune responses 24,25,26.

This study has certain limitations that should be taken into consideration, such as the lack of inflammatory analyses 48 hours after extubation and at the end of the SVT, which could provide pre- and post-test standards for comparison or late inflammation assessment in these patients.

In our opinion, the present study creates new possibilities for research into the SVT. The role of the inflammatory system in patients who undergo this process should be better explored and clarified.

We can conclude that there is a higher level of CRP in patients who failed the SVT than in patients who passed it and higher levels of IL-6 in patients who failed the SVT. Furthermore, higher TNFα, IL-6 and IL-8 levels are related to increased incidence of failure.

## AUTHOR CONTRIBUTIONS

Forgiarini SG was responsible for the project preparation, data collection, data analysis and manuscript writing. Rosa DP and Forgiarini LP were responsible for the project preparation and biochemical analyses. Teixeira C was responsible for the project preparation, data analysis and manuscript writing. Andrade CF was responsible for the project preparation and data analysis. Junior LA was responsible for the project preparation, data collection, data analysis, manuscript writing and submission. Felix EA and Friedman G were responsible for the project preparation, data analysis and manuscript writing.

## Figures and Tables

**Table 1:** Baseline characteristics of the patients.

Characteristics	n=54
Gender, male	32 (59%)
Age, years	70±18.02
Weight, kg	73.56±12.69
Height, cm	166.6±16.7
BMI	25.7±4.09
APACHE II score	22.5±6.6
Mechanical ventilation time, days	8.9±1.3
Underlying disease	
ARF	37 (68.6)
Sepsis	6 (11.1)
PO	11 (20.4)
Comorbidities	
Cardiovascular disease	10 (18.5)
Pulmonary disease	8 (14.8)
Other	27 (50)
No comorbidities	9 (16.7)

Data expressed as n (%) and mean±standard deviation. kg - kilograms; cm - inches; BMI - body mass index; ARF - acute respiratory failure; PO - postoperatively.

**Table 2:** Comparison of patients with successful SVT with patients who failed.

Variables	Success (34)	Failure (20)	*p*
APACHE II score	22.25±7	22.2±6.2	0.97
IRSB	53.97±24.9	46.45±14.03	0.03
MIP, cmH_2_O	48.02±16.5	46.45±14.03	0.73
pH	7.42±0.07	7.44±0.06	0.29
PaCO_2_, mmHg	37.6±8.3	42.4±18.1	0.18
PaO_2_, mmHg	122.4±32.9	117.1±39.1	0.59
PaO_2_/FiO_2_	310.5±78.6	301.3±87.7	0.69
Fluid balance, mL	3733.5±1959.5	4051.2±2980.4	0.63

Data expressed as n (%) and mean ± standard deviation. IRSB - index of rapid shallow breathing; MIP - maximal inspiratory pressure; cmH_2_O - centimetres of water; PaCO_2_ - partial pressure of arterial carbon dioxide; PaO_2_ - partial pressure of arterial oxygen.

**Table 3:** Evaluation of interleukins (IL) and C-reactive protein (CRP) in patients with SVT success and failure.

Variables	All	Success	Failure	*p*
**IL-1 pg.mL^-1^**	1.22±0.63	1.17±0.64	1.29±0.63	0.56
**IL-6 pg.mL^-1^**	1.91±0.35	1.85±0.35	2.06±0.33	0.03
**IL-8 pg.mL^-1^**	2.24±0.35	2.18±0.36	2.35±0.33	0.09
**IL-10 pg.mL^-1^**	1.65±0.31	1.60±0.22	1.75±0.41	0.08
**TNFα pg.mL^-1^**	1.35±0.31	1.27±0.38	1.46±0.37	0.07
**CRP mg.dL^-1^**	0.81±0.53	0.72±0.46	0.95±0.62	0.12

Data expressed as n (%) and mean±standard deviation.
